# The Role of Bronchoscopy in the Diagnosis and Management of Patients with SARS-Cov-2 Infection

**DOI:** 10.3390/diagnostics11101938

**Published:** 2021-10-19

**Authors:** Davide Biondini, Marco Damin, Martina Bonifazi, Elisabetta Cocconcelli, Umberto Semenzato, Paolo Spagnolo, Stefano Gasparini, Marina Saetta, Elisabetta Balestro

**Affiliations:** 1 Respiratory Disease Unit, Department of Cardiac, Thoracic, Vascular Sciences and Public Health, University of Padova, 35128 Padova, Italy; marco.damin@aopd.veneto.it (M.D.); elisabetta.cocconcellli@aopd.veneto.it (E.C.); umberto.semenzato@aopd.veneto.it (U.S.); paolo.spagnolo@unipd.it (P.S.); marina.saetta@unipd.it (M.S.); elisabetta.balestro@aopd.veneto.it (E.B.); 2 Pulmonary Disease Unit, Department of Internal Medicine, Azienda Ospedali Riuniti, 60126 Ancona, Italy; m.bonifazi@univpm.it (M.B.); s.gasparini@univpm.it (S.G.); 3 Department of Biomedical Sciences and Public Health, Università Politecnica Delle Marche, 60126 Ancona, Italy

**Keywords:** COVID-19, diagnostic strategies, bronchoscopy

## Abstract

Bronchoscopy has several major diagnostic and therapeutic indications in pulmonology. However, it is an aerosol-generating procedure that places healthcare providers at an increased risk of infection. Now more than ever, during the spread of the coronavirus disease 2019 (COVID-19) pandemic, the infectious risk during bronchoscopy is significantly raised, and for this reason its role in diagnostic management is debated. In this review, we summarized current evidence regarding the indications for bronchoscopy and the measures that should be applied to decrease risk exposure. Indeed, seeing the long-lasting period of the pandemic, resuming standard of care for all patients is required.

## 1. Introduction

In December 2019, the first cases of coronavirus disease 2019 (COVID-19) were reported in Wuhan, China; since then, the diffusion of the severe acute respiratory syndrome coronavirus type 2 (SARS-CoV-2) has rapidly spread reaching pandemic proportions. As of August 2021, nearly 200 million people have been infected and more than 4 million people have died of this disease worldwide [1].

One of the greatest difficulties in successfully managing the spread of the virus is asymptomatic virus shedding. Indeed, previous reports demonstrated that nearly 80% of documented cases have extremely mild symptoms, or even no clinical signs whatsoever, while still shedding the virus [2,3].

SARS-CoV-2 enters the human cells through contact with mucosal surfaces binding the angiotensin-converting enzyme 2 (ACE2) receptor, which is highly expressed on alveolar epithelial type II cells, and to a lesser degree in the epithelial cells of the upper airways (oral, nasal, and the pharynx) [4,5,6].

As a result, the gold standard diagnostic tests for detecting current SARS-CoV-2 infections are based on reverse transcriptase polymerase chain reaction (RT-PCR) with samples collected from the upper respiratory tract (nasopharyngeal nasal or oropharyngeal swabs) [7]. Indeed, in the prodromal phase, when the contagiousness is higher, the active viral replication of the virus can be localized and identified in the upper airways [8]. However, these tests do not have high sensitivity, ranging from 32 to 63% due to wrong handling of the specimen, sample collection during the late phase of the disease, or low viral load [9,10]. Among patients with clinical suspicion of COVID-19, with negative nasopharyngeal swabs, samples from the lower respiratory tract using bronchoscopy could increase sensitivity and help to achieve a correct diagnosis. Indeed, Wang and colleagues compared positive RT-PCR tests on different clinical specimens in patients with COVID-19 and showed that bronchoalveolar lavage fluid (BALF) was positive in 93% of cases, compared to sputum (72%), nasal (63%), and pharyngeal swab (32%) [10].

The aim of this review was first to analyze the role of bronchoscopy in patients with COVID-19 pneumonia, focusing in particular on its indication and utility for the management of suspected cases. Then we described the conduct of endoscopic procedures and the rules currently followed in the COVID era. Lastly, we then explored the future perspective of interventional pulmonology activity.

## 2. Role of Bronchoscopy in the Diagnostic Work-up of COVID-19 Infection

The role of bronchoscopy in COVID-19 is still a matter of vivid debate, in particular given the high contagious risk of the procedure. The reasons are mostly due to the significant amounts of droplets that contaminate the indoor equipment and the procedure room’s air, the increased pressures used to oxygenate or ventilate the patients with respiratory failure, and especially the close contact between the medical personnel involved in the procedure and the patient [11].

Although many scientific societies have issued guidelines in order to reduce heterogeneity in clinical practice [12], the scientific background supporting bronchoscopy is poor and mainly composed of case series [13,14,15].

In the National Institute of Health COVID-19 treatment guidelines panel (last updated on August 21) [7], bronchoscopy with bronchoalveolar lavage (BAL) of the lower respiratory tract is only indicated in patients with clinical signs and symptoms consistent with COVID-19 pneumonia but a negative upper respiratory tract swab in order to confirm or exclude a diagnosis of COVID-19, even though they suggested that endotracheal aspirates should be preferred over BAL whenever possible.

Various types of COVID-19 diagnostic guidance have been proposed by several Endoscopic and Pulmonologists Societies during the pandemic [11,16,17,18,19,20], which recommended bronchoscopy in suspected COVID-19 cases for typical clinical and radiological features but with a concomitant negative oropharyngeal swab. However, it is not clearly specified how many negative samples are required to support the indication.

Ora J. and co-workers investigated this issue and suggested that three negative swabs, performed on three consecutive days, along with negative serology, despite highly suggestive clinical features and a computed tomography (CT) scan, can safely rule out the SARS-CoV-2 infection [21]. Indeed, all BALF obtained in this population were negative for SARS-CoV-2 virus, but showed, in nearly half of them, a different isolation, hence allowing an alternative diagnosis [21].

In another study that evaluated a population with similar clinical characteristics, but with only two consecutive negative nasopharyngeal swabs, a low diagnostic yield of BALF for detecting SARS-CoV-2 virus (36%) was reported [22].

These results are in contrast with other studies, in which BALF helped in determining COVID-19 diagnosis with higher rates reported, ranging from 55 to 93% [9,13,21]. This could be in part explained by a lower number (one or two) of oro/nasopharyngeal swabs previously performed.

Overall, as most of the studies showed, one of the leading roles of bronchoscopy in this context is to identify potential alternative infections or coinfections, in particular in immunosuppressed patients. Interestingly, recent studies reported alternative infectious diseases in up to 65% of patients, causing a change in the pharmacological care of the disease [13,21].

The diagnostic limits of oro/nasopharyngeal swabs could be offset by chest CT features, which showed a sensitivity of 97% in suspected COVID-19 cases [9]; however, to date, the lack of standardized diagnostic algorithms including clinical and radiologic features together with RT-PCR results might be the reason of requesting not properly further invasive procedure such as bronchoscopy.

## 3. Role of Bronchoscopy in the Management of COVID-19 Infection

During the pandemic, patient management varied based on the severity of respiratory failure. Indeed, when a low-flow oxygen supplementation through nasal cannula or face mask was required, patients were managed in a low-intensity medical care (LIMC) ward, such as internal medicine or infectious disease unit. Conversely, when these strategies were not sufficient and high-flow nasal cannula (HFNC) or invasive/non-invasive ventilation were needed, patients were admitted to high-intensity medical care (HIMC) wards, such as a Respiratory Intensive Care Unit (RICU) or ICU [23]. In the setting of critically ill patients, bronchoscopy had a particularly important role. Indeed, in those who require non-invasive or invasive ventilation, several complications may occur, thus hampering the effectiveness of ventilation. For instance, lobar atelectasis is a potential acute complication of severe COVID-19 that is generally determined by the presence of mucus plugs and is associated with poor outcomes [24].

Similarly, hemoptysis or bloody mucus from the lower respiratory tract, which may complicate the most advanced and severe forms of COVID-19 with a high mortality rate [25], is another cause of ineffective ventilation because of complete bronchial obstruction. Conversely, the presence of diffuse mucosal hyperemia is typical of an earlier phase of COVID-19 that indicates a potentially reversible acute inflammation associated with reduced in-hospital mortality rates [26].

Mechanically ventilated patients with COVID-19 are prone to develop ventilator-associated pneumonia (VAP) that could be unrecognized because of its clinical and radiographic similarity to COVID-19. VAP has an incidence ranging from 29 up to 80% [27,28] in these patients, with a hazard ratio of 2.1 compared to that in non-COVID-19 patients [29]. Such high incidence may be due to several factors, such as the treatment-associated immune impairment and prolonged mechanical ventilation or sedation. In those cases, bronchoscopy might help to formulate the correct diagnosis.

With regard to superimposed infections, fungal co-infection has an incidence of up to 34% in COVID-19 patients hospitalized in the ICU. In this case, COVID-19 associated pulmonary aspergillosis (CAPA) has a mortality rate of 36% [30]. Similar to COVID-19, pulmonary aspergillosis may manifest with fever, dyspnea, or respiratory failure and pulmonary infiltrates. Therefore, the diagnosis of CAPA is based on microbiological criteria, and BAL analysis is an important tool in this regard [30].

**Figure 1:**
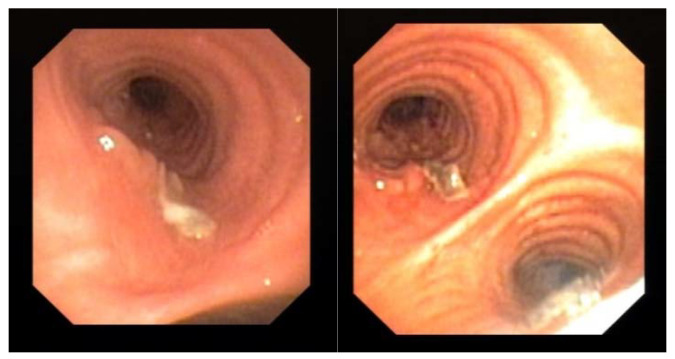
Evidence of mucosal infiltration and pseudomembranes in the left main bronchus, in a COVID-19 associated pulmonary aspergillosis (CAPA). The patient underwent a bronchial biopsy for the histological diagnosis.

Endoscopic examination through bronchoscopy may also reveal the presence of lesions (i.e., epithelial plaques, pseudomembranes, or ulceration of the bronchial mucosa) that may not be detectable by radiologic exams. We observed these findings in one patient with COVID-19 (Figure 1). These lesions resembled lung cancer infiltration and only bronchial biopsy allowed a correct diagnosis of CAPA.

In HIMC wards, where non-invasive/invasive ventilation is performed, pneumomediastinum is an additional frequent complication, despite the use of protective ventilation. Indeed, in a study that compared the incidence of pneumomediastinum in ARDS secondary to COVID-19 to that of other causes, the authors observed a higher incidence of pneumomediastinum in COVID-19 ARDS patients (13.6% vs. 1.9%, *p* < 0.001) [31]. In the management of this complication, bronchoscopy can identify the presence of bronchial or tracheal injury.

Apart from the complications of COVID-19 mentioned above, bronchoscopy has a role in the management of patients with conditions not related to COVID-19.

During the pandemic, most of the elective bronchoscopies have been suspended or rescheduled; indeed patients have been stratified according to emergent or urgent indications, as defined by the American College of Chest Physicians and the American Association for Bronchology and Interventional Pulmonology (CHEST/AABIP) [24].

Emergent indications were life-saving procedures, which could not be delayed, such as moderate symptomatic or worsening tracheal/bronchial stenosis, symptomatic central airway obstruction, and migrated stent [24] (Table 1).

**Figure 2:**
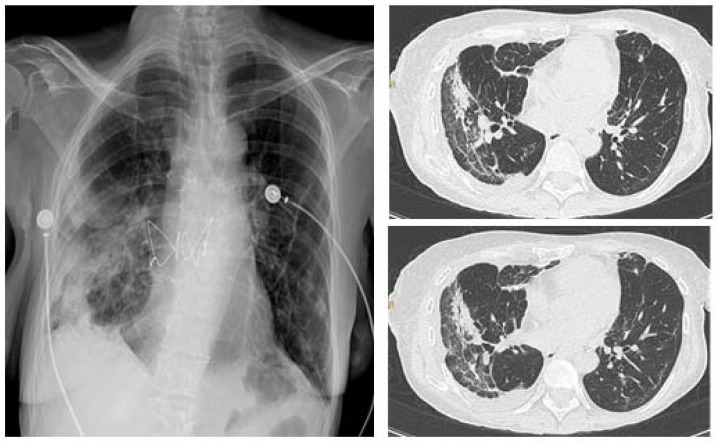
Chest X-ray (CXR) (**left**), CT scan (**right**) of a lung transplanted patient who was infected by SARS-CoV-2 during the recovery after transplant. The patient underwent a tranbronchial bronchial biopsy and was histologically diagnosed with SARS-CoV-2 pneumonia.

Urgent indications include lung cancer diagnosis (lung mass or mediastinal/hilar lymphadenopathy), foreign body aspiration, and suspected pulmonary infection in immunocompromised patients (Table 1). In the latter case, early bronchoscopy is essential because it may rule out the presence of a co-infection, thus allowing timely treatment. Moreover, in patients that underwent lung transplantation, transbronchial biopsies have an important role in identifying the presence of acute cellular rejection. Figure 2 shows the CT scan of a transplanted patient who was infected by SARS-CoV-2 during the recovery after lung transplant. The persistence of consolidations in the right lower lobe despite treatment for COVID-19 suggested a possible acute cellular rejection. However, the patient was histologically diagnosed with SARS-CoV-2 pneumonia.

**Figure 3:**
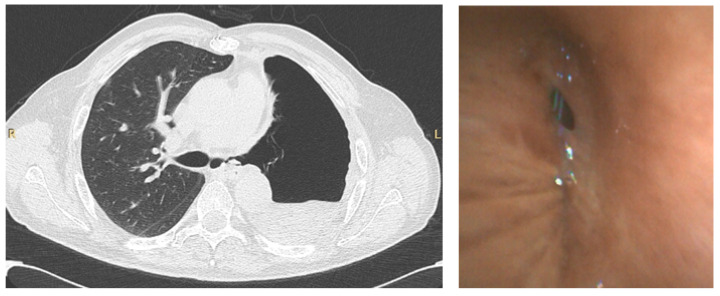
CT scan (**left**) of a patient that underwent left pneumonectomy, showing a possible left bronchopleural fistula, in a COVID-19 positive patient, confirmed by bronchoscopy (**right**) using single-use flexible bronchoscope.

Bronchoscopy during the pandemic was also useful in other scenarios. In our institution, apart from the emergent/urgent indications mentioned above, we performed bronchoscopy in a patient with COVID-19 that underwent a left pneumonectomy for lung cancer, to investigate the presence of a bronchopleural fistula, which was confirmed, in order to decide the best therapeutic intervention (Figure 3).

In addition, BAL may have a prognostic role; indeed, the identification of an extensive alveolitis in COVID-19 patients, particularly in the presence of high levels of IL6 and IL8, correlates with the severity of the disease and predict clinical outcomes [32].

As shown, bronchoscopy, and especially BAL, may have multiple indications. However, during the pandemic peak, patients had to be carefully selected not only for the risk of contagion for the healthcare personnel, but also for the patients given the risk of further deteriorating respiratory failure [33].

## 4. How to Perform Bronchoscopy

To protect providers and patients alike, approaches to minimize the risk of exposure while preserving the ability to perform appropriate aerosolizing procedures have been proposed by several guidelines from different societies of pulmonologists (American Association for Bronchology and Interventional Pulmonology, AABIP; Respiratory branch Chinese Medical Association, CMA; German Respiratory Society, DGP; Spanish society of pneumology and thoracic surgery, SEPAR; Italian society of pneumonology, AIPO) and can be summarized as follows [11,16,17,18,19,20]:

- patient questionnaires regarding fever, recent travel, or exposure to any COVID-19 positive patients, and symptom screening should be considered.
- the procedure should be performed in a negative pressure room which requires a minimum of 12 total air exchanges per hour in order to provide adequate dilution and exhaust of contaminated air. At this rate, after 23 min 99% of particles will be exchanged and, after 35 min, the percentage reaches 99.9% [34].
- to minimize the risk of transmission during bronchoscopy in COVID-19 patients, a reusable acrylic barrier enclosure could be used during standard intubation, and utilizing disposable drapes to create a contained tent immediately around the patient [35].
- the presence of the staff should be reduced (bronchoscopist, bronchoscopy assistant, anesthesia team if necessary) to a main team, who carry out all the interventions of the day to minimize staff exposure. No observers, students, apprentices, or trainees should be in the examination room.
- to protect the personnel, powered air-purifying respirators (PAPRs) or N95 respirators/FFP3 masks should be worn; eyes should be protected by disposable safety glasses and/or a face shield, as well as the use of disposable gowns, gloves, and cap.
- for anesthesia, atomized lidocaine should be avoided and it should aggressively reduce cough. Moreover, standard induction of anesthesia may be replaced by rapid-sequence induction, preferring an endotracheal tube over a supraglottic airway.
- rigid bronchoscopy should be avoided, and for flexible bronchoscopy transnasal access is preferred, with the additional use of a slotted mouth and nose protector for the patient, or even a box plexiglass can be used.
- if ventilation is needed during general anesthesia, jet ventilation should be avoided, when possible, with closed ventilation systems (tube, laryngeal mask).
- when reusable bronchoscopes are used, sterilization should be considered instead of high-level disinfection, according to the updated recommendation for reprocessing of the Food and Drug Administration (FDA). When the support for immediate reprocessing is lacking, providers should use single-use bronchoscopes.

Post-procedural considerations are equally important for the safety of healthcare personnel and prevention of nosocomial transmission of COVID-19. According to van Doremalen N. and co-authors, SARS-CoV-2 can remain aerosolized for up to 3 h and is viable on plastic and stainless-steel surfaces for up to 72 h [36]. Following bronchoscopy, the patient should be recovered according to local protocol. All staff involved should then take off the personal protective equipment (PPE) and perform hand hygiene. All horizontal and work surfaces, video monitors, and hardware should then be disinfected with Environmental Protection Agency (EPA)-approved cleaners. Disposable equipment should be discarded, and medical waste collected with routine biohazardous waste [37].

## 5. Single-Use Bronchoscopes

Bronchoscopes are medical devices at a high risk of residual microbial contamination due to their long and relatively small working channel size when compared with devices such as gastroscopes. Indeed, device contamination can also be documented, even if rarely, with high-level disinfection and sterilization, which does not always avoid cross-contamination alone [38].

In addition, multidrug resistance (MDR) pathogens infections are now increased, predisposing outbreaks of bronchoscopy-related transmission of multiple drug resistance pathogens like *Pseudomonas aeruginosa* and *Klebsiella pneumoniae* [39].

To minimize these risks, in the last years, disposable bronchoscopes have been produced and released on the market by several companies (Ambu, Glidescope, Olympus, Pentax, Boston Scientific, Broncoflex, Vathin, etc.), and their use is limited to the local resources and availability of the country.

Clearly, single-use flexible bronchoscopes (SUFBs) are sterile, opened and used only for a single patient, avoiding the risk of any type of bronchoscope cross-contamination from one patient to the other, as well as the risk for the healthcare workers during transporting or reprocessing/sterilization.

In any suspected or confirmed COVID-19 patient, the AABIP [16] recommends SUFBs as first line because it minimizes the risk of contamination; moreover, the portable screens are easy to clean and they do not need any reprocessing after using it [40].

The cost effectiveness of SUFBs versus reusable flexible bronchoscopes (RFBs) depends on the volume of activity; indeed it has been reported that RFBs become less cost-effective than SUFBs [41] in endoscopy or intensive care units which perform a small number of interventions, while RFBs may become more affordable and therefore are preferred with increased activity/demand [42]. However, this could be variable depending on the maintenance and repair costs of RFBs, which are elevated. Interestingly, nearly half of the cost of bronchoscope repair can be attributed to preventable damage, as a common mistake of unsheathing a biopsy needle within a working channel [43]. Therefore, a careful knowledge of the risk of such mistakes and how to avoid them could impressively drop the repair cost [44].

A recent review [45] that took into account 16 studies performed in endoscopy and intensive care units showed that the cost per use for a SUFBs was slightly lower than RFBs, and this gap augmented whether it was considered the potential costs of treatment of infection due to contaminated RFBs. However, this study showed some limitations, because the cost effectiveness of SUFBs in endoscopy units is not comparable to that in intensive care unit, and these data should be further validated considering the same working setting. Certainly, mixed equipment is the more realistic alternative at the moment instead of considering one the replacement of the other.

## 6. Future Evidence and Perspectives: “The New Routine”

Over the course of these months, the approach to the patient has changed dramatically in nearly every country worldwide in almost each spectrum of care.

During the worst phases of the COVID-19 pandemic, guidelines suggested to postpone elective procedure focusing on urgent/emergent procedures, basically to minimize the risk of infection for the healthcare professionals, as previously mentioned.

However, after more than 21 months from the beginning of SARS-CoV-2 spread, we learned to cohabit with virus spread since it is still present, and it is not predictable whether it will be eradicated or not. In the meanwhile, the time has come to move on and consider all the new protocols used in the bronchoscopic units as the daily routine from now on, in order to come back as close as possible to the number of procedures as in the pre-COVID19 era in all the interventional pneumology centers; indeed, it is unrealistic to continue the delay of certain procedures, even the elective ones.

The level of community spread and individual risk is unknown, people can be healthy carriers, and the not optimal sensitivity of molecular tests leads to considering every patient in the endoscopic rooms as a potential carrier.

As the COVID-19 testing capabilities have improved with a shortened turnaround time and healthcare facilities are gradually allowing elective procedures, pre-procedural COVID-19 testing should be obtained and verified along with a review of epidemiological and clinical markers of the active disease, ideally closely timed before the planned procedure [33].

Ultimately, the decision to implement pre-procedural COVID-19 testing should consider testing capability, availability, and regional disease prevalence.

Regardless, standard precautions should be taken for all healthcare workers, in the bronchoscopy room, to minimize transmission given the false negativity rate with testing. In particular, gowns, gloves, eye protection or face shields should be worn along with a fitted National Institute for Occupational Safety and Health (NIOSH)-certified N95 mask or powered air-purifying respirator (PAPR) as indicated in various guidelines [11,16,17,18,19,20]. Moreover, regarding bronchoscopy for outpatients, they should be asked about symptoms (fever with >37.5 °C, cough, sore throat, or respiratory problems in the past 14 days), contacts with a suspicious or confirmed case of COVID-19, and travel history. Once scheduled, the patient should be recontacted and also screened the day before the planned procedure.

In this rapidly changing public health environment, we also acknowledge that best practices may vary among hospitals based on local resources, expertise, patient populations and despite the fact that the continual updating of recommendations from major health organizations such as the US Centers for Disease Control and Prevention (CDC; www.cdc.gov, accessed on 16 August 2021) and the World Health Organization (WHO; www.who.int, accessed on 16 August 2021) is required.

Currently, the balance between timely care and risk of exposure to SARS-CoV-2 is a pressing matter in the endoscopic unit. In particular, timely interventions for patients that need a diagnostic procedure (e.g., lung cancer diagnosis) must be required and cannot be further delayed. Indeed, recently, Pasello G. et al. [46] investigated this issue, evaluating how the COVID-19 pandemic impacts on the integrated care pathways for lung cancer diagnosis and treatment, comparing a period before and a period during the pandemic. Interestingly, in this center the number of bronchoscopic procedures that were prioritized on the oncologic patients increased. On the contrary, the time between the first pulmonologist evaluation and the first oncologic visit was slightly prolonged due to a longer duration of the diagnostic pathway. Moreover, as previously reported [47], the activities of pathology departments were affected by the reduction on-site of technical and administrative personnel, because they were involved in the post-mortem examination of COVID-19-positive patients.

Since the beginning of the pandemic, the risk of viral transmission to healthcare workers during bronchoscopy has been considered very high. However, it has not been deeply investigated since the recent commentary by Saha B.K. and Chenna P. [48]. Indeed, they have summarized seven cohort studies that assessed the risk of COVID-19 transmission during bronchoscopy among bronchoscopists and other healthcare workers in mechanically ventilated patients. Considering these studies together, a total of 650 patients were included who underwent approximately 1200 bronchoscopies. In particular, 60 bronchoscopists were involved with an average of 16.8 exams each. Of note, only two of them were infected by SARS-CoV-2, while no infections were observed among bedside nurses, respiratory therapists, or technicians. A limitation of this analysis is the significant heterogeneity in terms of methods used to identify the infection; however, it should be noted that all bronchoscopies were performed following the guidelines provided by the WHO, CDC and other professional societies, and a disposable bronchoscope was used in the majority of the studies. In addition, because only bronchoscopies in ICU were considered; further studies are required to assess the safety and feasibility of procedures performed in a routine endoscopic suite. Overall, these studies strongly support the use of bronchoscopy in COVID-19 patients whenever clinically indicated. Indeed, when appropriate infection control precautions are taken and healthcare workers use appropriate personal protective equipment (PPE), the risk of infection appears to be very low [48].

Following SARS-CoV-2 infection, patients may experience long-term health consequences, including, among others, persistent radiographic abnormalities (organizing pneumonia, fibrotic-like changes, bronchiectasis) [49,50,51,52,53] and post intubation or post tracheostomy complications, such as tracheal stenosis or granuloma formation [54]. Bronchoscopy may also play a role in the diagnosis and management of these long-term complications.

Pulmonary fibrosis secondary to COVID-19 is a major concern, and can be due to a number of triggers, including viral infection, ARDS and “cytokine storm”, and mechanical ventilation [49]. In the acute phase of COVID-19, the predominant pathological pattern of lung lesions is diffuse alveolar damage, along with hyaline membranes, pneumocyte atypical hyperplasia and platelet–fibrin thrombi in small arterial vessels [55]. Moreover, fibrotic lung parenchymal damage, characterized by fibroblast proliferation, airspace obliteration, and micro-honeycombing, has been observed in the acute phase in deceased subjects with COVID-19 in autoptic series as well as in a cohort where tissue had been obtained from transbronchial lung cryobiopsy within 30 min of death [55,56,57]. However, pathological data regarding subsequent phases of post-COVID-19 lung lesions are lacking. Post-COVID-19 interstitial lung disease (ILD) has also been described in radiological follow-up studies. In the first weeks after the infection, organizing pneumonia (OP) is the prevailing pattern on CT [50]; by contrast, in cohorts with longer follow-up (6 and 12 months), interstitial lung abnormalities (ILAs) and established pulmonary fibrosis tend to prevail [52,53]. OP and pulmonary fibrosis have different clinical behavior, rate of progression, response to treatment, and prognosis; therefore, differentiating between the two, which may not be straightforward on imaging, is critically important. In patients with ILD without typical (i.e., diagnostic) radiological patterns, surgical lung biopsy (SLB) remains the diagnostic gold standard [58]. However, because of the substantial morbidity and mortality of SLB, particularly in nonelective procedures, transbronchial lung biopsy and transbronchial lung cryobiopsy may have a role in the diagnostic work-up of patients with COVID-related ILD.

Among severe COVID-19 patients, endotracheal intubation and mechanical ventilation are often necessary. Tracheal stenosis, a well-known long-term complication of intubation [59,60], has also recently been described in COVID-19 intubated patients [54]. Bronchoscopy is a useful tool for the diagnosis and the management of this sequela. In fact, apart from the surgical approach, these patients can also be treated with bronchoscopy techniques, such as lasers, balloon dilatation, electrocauterization, cryotherapy, argon plasma coagulation, and stent placement [61,62]. In intubated patients undergoing tracheostomy, bronchoscopy might be useful for the management of decannulation and for the diagnosis of granuloma formation [63]. Thus, in a pandemic context, in which a large number of patients underwent endotracheal intubation (and eventually tracheostomy), physicians must be aware of the possible long-term consequences of this therapeutic intervention, in order to promptly diagnose and treat them.

In conclusion, bronchoscopy during the pandemic peak has been considered a procedure with a high contagious risk for the healthcare workers. As a result, a risk stratification for performing the procedure has been put in place in order to delay the less urgent procedures. However, it has become increasingly clear that bronchoscopy can be performed safely in all the scenarios in which it is clinically indicated. Moreover, if protective equipment is correctly used, the rate of infection of the healthcare personnel reported was low. Therefore, we believe that bronchoscopy, with adequate protection and infection control, should no longer be postponed, and all the protection protocols for the procedure should become routine in the endoscopic suites from now on.

## Figures and Tables

**Table 1:** Indications for bronchoscopy in suspected COVID-19 and indication for urgent or emergent in confirmed COVID-19 during the pandemic peak.

Suspected COVID-19	Confirmed COVID-19: Emergent Indication	Confirmed COVID-19: Urgent Indication
Confirm or exclude COVID-19 in those with a negative upper respiratory tract swab, but clinical signs and symptoms consistent for COVID-19 pneumonia [7]	Moderate symptomatic or worsened tracheal/bronchial stenosis; migrated stent	Lung cancer diagnosis (lung mass or mediastinal/hilar lymphadenopathy)
Confirm suspected COVID-19 cases with a negative upper respiratory tract swab, but typical clinical and radiological features [11,15,16,17,18,19,20]	Symptomatic central airway obstruction (i.e., due to mucus plug) or lobar atelectasis	Foreign object aspiration
Confirm or exclude COVID-19 in those with a negative upper respiratory tract swab and clinical signs and symptoms possible for COVID-19 pneumonia, but an alternative diagnosis could also be considered [7]	Hemoptysis or bloody mucus from the lower respiratory tract	Suspected concomitant pulmonary infection in immunosuppressed patients (i.e., fungal co-infection)
